# First report of multidrug-resistant *Salmonella* Infantis in broiler litter in Tolima, Colombia

**DOI:** 10.14202/vetworld.2022.1557-1565

**Published:** 2022-06-28

**Authors:** Mayra A. Bonilla-Caballero, María P. Lozano-Puentes, María A. Ospina, Maryeimy Varón-López

**Affiliations:** Department of Biology, Research Group on Plant and Microbial Biotechnology - GEBIUT, Faculty of Sciences, University of Tolima, PO Box 730006299, Ibagué, Colombia

**Keywords:** antibiotics, cefotaxime, poultry, *Salmonella*

## Abstract

**Background and Aim:**

*Salmonella* has been identified as one of the most widely distributed zoonotic pathogens in broiler litter. Multidrug-resistant strains have been isolated from salmonellosis outbreaks, compromising the success of their treatment. This study aimed to isolate and identify *Salmonella* spp. serovars in healthy broiler litter in Tolima (Colombia), determine their resistance to different antimicrobials, and detect genes associated with b-lactam resistance that could be useful to control *Salmonella* spp. in poultry.

**Materials and Methods:**

In total, 45 broiler litter samples were collected. *Salmonella* spp. was isolated and identified using selective and differential culture media and biochemical tests. Molecular confirmation of the pathogen was performed with the invA gene and serotyping by Kauffman–White scheme. Antimicrobial susceptibility to 15 antibiotics was determined by Kirby–Bauer method. In cefotaxime-resistant strains, *blaCTX-M*-F, *blaCTX-M-1*, *blaCMY*, and *blaTEM* genes were evaluated by polymerase chain reaction (PCR).

**Results:**

In total, 817 presumptive strains were obtained from xylose lysine deoxycholate and *Salmonella* *Shigella* agars and subcultured on xylose-lysine-tergitol 4 and MacConkey agars, from which 150 strains were isolated; 29 of these strains were presumptive for *Salmonella* spp. after performing biochemical tests and 16 were confirmed by PCR as *Salmonella* Infantis (15) and Gallinarum (1). All strains were found to be multiresistant to antibiotics, showing three different profiles and isolates resistant to cefotaxime, and the *blaCTX-M* gene was detected.

**Conclusion:**

This is the first study to isolate *S*. Infantis from broiler litter in Colombia. All isolates exhibited resistance to the evaluated antimicrobials, suggesting the misuse of antimicrobials in small- and medium-sized poultry farms. The presence of *Salmonella enterica* serovar Infantis is a public health problem. Thus, regular monitoring of poultry litter is recommended, as these bacteria can be transmitted to humans through animal products or contaminated environments.

## Introduction

*Salmonella* is a zoonotic pathogen with a wide range of animal and human hosts; it has been identified as a genus of global public health importance and the leading cause of foodborne illnesses responsible for thousands of deaths worldwide [1, 2]. This microorganism is one of the most prevalent pathogens in broiler litter (substrate that covers the floor of the shed with a high microbiological load) and can be transmitted from one production cycle to another. This can happen when litter is used in several consecutive batches and without adequate treatments to colonize the digestive tract of broilers, as broilers typically are in direct contact with litter and consume it [3–5]. In addition, residues of antibiotics fed to broilers can be found in the litter. When incompletely metabolized, 60–90% of antibiotics can be excreted in the broiler’s excreta [6, 7]. Different studies have shown that *Salmonella*, especially serotypes Infantis, Gallinarum, Kentucky, and Saintpaul [8–10] isolated in poultry environments, has high resistance to b-lactams, associated with the presence of *blaCTX-M*, *blaCTX-M-1*, *blaCMY*, and *blaTEM* genes [11–14].

Identifying propagation sources of pathogens on farms, such as poultry litter has been deemed vital for controlling microorganisms such as *Salmonella*. Monitoring poultry flocks are the first step to reduce the transmission of pathogens to humans and the antimicrobial resistance of these pathogens and indirectly increases disease treatment options [15].

Therefore, this study aimed to isolate and identify *Salmonella* spp. serovars in litter from healthy broilers in the department of Tolima (Colombia), estimate resistance to different antimicrobials, and detect genes associated with b-lactam resistance.

## Materials and Methods

### Ethical approval

The experiment was approved by the Animal Ethics Committee of University of Tolima, Colombia. Farms were selected after consideration of the willingness of the farmers. During the collection of samples, written consent was taken from each of the farm owners or farm managers.

### Study period and location

The study was conducted from June to December 2020. The experiment was carried out on three broiler farms located in the department of Tolima, Colombia.

### Sample collection and preparation

In total, 45 rice husk litter samples (each sample comprised of 10 subsamples) were collected from broiler farms (1200–22,000 broilers per house). Fifteen samplings were performed on 0 (before the broilers arrived), 3, 15, 28, and 43 days of the production cycle. Samples were collected from the entire depth of the litter without scratching the original soil surface and placed in sterile Ziploc bags. After collection, 10 g of a combination of litter samples was transferred to the laboratory for immediate analysis and placed into sterilized stomacher bags containing 90 mL of buffered peptone water (BPW) (Acumedia, Lansing, Michigan).

The sample size was calculated using the following formula [16]:


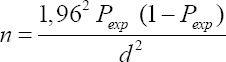


This formula was selected as the best fit for this study, as no studies of *Salmonella* spp. in poultry litter in Tolima were carried out before; therefore, no other formulas were available for this specific case. *n* is the required sample size, *P_exp_* is the expected prevalence, and *d*^2^ is the desired absolute precision. The confidence level was 95%, the precision level was 5%, and the expected prevalence was 3. Applying this formula, the minimum number of samples was 44.71. Therefore, in total, 45 samples were collected.

Sample selection was carried out through the non-probabilistic method, as it depended on the willingness of the farm owners to participate in this study. Before sampling, a survey was conducted; variables, such as the number of birds; biosecurity measures; material of the litter; reuse and sanitation of the litter; management of physicochemical factors; control of rodents, wild birds, and insects; and management and sanitary status of the animals, were included in the survey.

### Isolation of *Salmonella*

All samples were pre-enriched in 9:1 BPW (Acumedia) for 18–24 h before transport to the Laboratory of Microbiology and Mycorrhizae for further analysis. All samples were subjected to selective culture and biochemical testing for the presence of *Salmonella enterica*. The pre-enriched samples were inoculated in Rappaport-Vassiliadis broth (Oxoid; Basingstoke, Hampshire, England) at 41°C for 24 h. The samples were subsequently cultured in xylose lysine deoxycholate (XLD) (Oxoid) and *Salmonella*-*Shigella* (SS) (Oxoid) agars at 37°C. Subsequently, 817 colonies were taken for subculture in xylose-lysine-tergitol 4 (XLT4) (Condalab; Madrid, Spain) and MacConkey agars (MCA; Acumedia, Michigan, US) at 37°C overnight for 24 h.

### Identification of *Salmonella* spp.

Biochemical identification was made using Gram staining and oxidase test. All isolates that were Gram-positive and/or oxidase-positive were discarded. The other isolates were biochemically tested using triple sugar iron agar (Oxoid), Simmons citrate (Oxoid), lysine iron agar (Acumedia), and sulfide indole motility agar (Merck, Darmstadt, Germany), and urease.

### DNA extraction

DNA extraction of presumptive *Salmonella* spp. strains was done by boiling method [17]. The quantity and purity were assessed using NanoDrop and Qubit, and DNA quality was checked on a 1.5% (w/v) agarose gel and visualized using an ultraviolet (UV)–visible spectrophotometer (Cole-Parmer UV Transilluminator, Vernon Hills, USA). DNA was stored at −20°C until use.

### Polymerase chain reaction (PCR)

All isolates were confirmed by PCR by the amplification of the *invA* gene. *S*. *enterica* subsp. *enterica* serovar Enteritidis ATCC^®^ 13076 strain (Thermo Scientific, United Kingdom) was used as a positive control. Amplification was performed in a T-100™ thermocycler (Bio-Rad, USA). All PCRs were performed in a total volume of 12.5 mL consisting of 6.25 mL of 2× PCR MasterMix (Corpogen, Bogota, Colombia), 2.5 mL DNA template, and 0.65 mL of each primer brought to 2.5 mL using DNA/RNA-free water. PCR and thermal conditions were performed according to the referenced authors (Supplementary data not shown). Amplicons were then visualized on a 1% agarose gel by electrophoresis (PowerPac™ HC, Bio-Rad) using 1 kb DNA ladder Load Ready™ (Amplyus, USA). The gel was stained with HydraGreen™ (ACTGene, USA) and visualized under the E-Gel™ Imager System with UV Light Base (Thermo Fisher Scientific, Waltham, MA).

### Serotyping

Serotyping of *Salmonella* spp. strains was carried out at the Colombian Agricultural Institute (ICA), following the Kauffmann–White scheme, to determine the O (somatic), Vi (capsular), and H (flagellar) antigens [18].

### Antibiotic sensitivity assay

Isolated *Salmonella* spp. were subjected to an antimicrobial sensitivity test by the Kirby–Bauer disk diffusion method [19]. Overnight grown bacterial inocula were adjusted to the 0.5 McFarland standard, swabbed on pre-incubated Mueller-Hinton agar (MHA) plates by a sterile cotton swab, and subsequently left for 10–15 min to dry. Afterward, standard antibiotic disks (Oxoid) were placed on MHA plates with sterile forceps, and aerobic incubation took place at 37°C for 24 h. After incubation, the organisms were categorized as “resistant” or “susceptible” based on the diameter of their zone of inhibition according to the Clinical and Laboratory Standards Institute guidelines [20]. Fifteen antibiotics were used: Ampicillin-sulbactam (10 mg), amoxicillin (10 mg), gentamicin (10 mg), ciprofloxacin (10 mg), cefotaxime (30 mg), erythromycin (15 mg), nalidixic acid (30 mg), penicillin (10 mg), trimethoprim-sulfamethoxazole (25 mg), tetracycline (30 mg), ceftiofur (30 mg), enrofloxacin (5 mg), colistin (10 mg), streptomycin (10 mg), and doxycycline (30 mg). Strains were considered multiresistant when they were determined to be resistant to three or more groups of antibiotics.

### PCR detection of extended-spectrum β-lactamase (ESBL) resistance genes

The presence of ESBL resistance genes was determined by PCR using gene-specific primers (Supplementary data not shown). DNA extracted from the isolates was used as the template for the PCR assay conducted under the above-described conditions. PCR products were validated by electrophoresis and sequenced at Macrogen (South Korea) for the confirmation of target genes. Sequence alignment was performed using the Basic Local Alignment Search Tool.

### Statistical analysis

Microsoft Excel (Microsoft Corporation, USA) data analysis tools were used to develop descriptive statistics.

## Results

### Isolation of *Salmonella* spp.

In total, 817 strains were obtained from XLD and SS agars, of which, after culture in MacConkey and XLT4 agars, 150 presumptive strains for *Salmonella* were determined. After identification by biochemical tests, 29 presumptive isolates were acquired, and all came from the same litter sample. The isolates were molecularly confirmed with the *invA* gene, which was present in 16 isolates, to which the serotyping test was performed at the ICA. Fifteen strains corresponded to *Salmonella* Infantis and one corresponded to *Salmonella* Gallinarum (Table-1).

**Table 1:** Results of the microbiological and molecular tests carried out on the isolated strains of *Salmonella* spp.

Strain	Biochemical confirmation							Molecular confirmation (*InvA*)	Serotyping	Phenotypic resistance	Genotypic resistance

	Simmons citrate	TSI	LIA	SIM		Urea	SH_2_ production				

				Motility	Indole						
FCS1*	+	K/A	±	+	-	−	+	+	*S*. Infantis	CIP, GM, CTX, NA, TE, E, P, CTF, DO, S	*bla* CTXM-F
FCS2*	+	K/A	±	+	−	−	+	+	*S.* Infantis	CIP, GM, CTX, NA, TE, E, P, CTF, S	*bla* CTXM-F
FCS3*	+	K/A	±	+	−	−	+	+	*S*. Infantis	CIP, GM, CTX, NA, TE, E, P, CTF, DO, S	*bla* CTXM-F
FCS4*	+	K/A	±	+	−	−	+	+	*S*. Infantis	CIP, GM, CTX, NA, TE, E, P, CTF, DO, S	*bla* CTXM-F
FCS5*	+	K/A	±	+	−	−	+	+	*S*. Infantis	CIP, GM, CTX, NA, TE, E, P, CTF, S	*bla* CTXM-F
FCS6*	+	K/A	±	+	−	−	+	+	*S*. Infantis	CIP, GM, CTX, NA, TE, E, P, CTF, DO, S	*bla* CTXM-F
FCS7*	+	K/A	±	+	−	−	+	+	*S*. Infantis	CIP, GM, CTX, NA, TE, E, P, CTF, DO, S	*bla* CTXM-F
FCS8*	+	K/A	±	+	−	−	+	+	*S*. Infantis	CIP, GM, CTX, NA, TE, E, P, CTF, DO, S	*bla* CTXM-F
FCS9*	+	K/A	±	+	−	−	+	+	*S*. Infantis	CIP, GM, CTX, NA, TE, E, P, CTF, S	*bla* CTXM-F
FCS10*	+	K/A	±	+	−	−	+	+	*S*. Infantis	CIP, GM, CTX, NA, TE, E, P, CTF, S	*bla* CTXM-F
FCS11*	+	K/A	±	+	−	−	+	+	*S.* Infantis	CIP, GM, CTX, NA, TE, E, P, CTF, DO, S	*bla* CTXM-F
FCS12*	+	K/A	±	−	−	−	−	+	*S*. Gallinarum	GM, CTX, NA, TE, E, P, CTF, S	*bla* CTXM-F
FCS13*	+	K/A	±	+	−	−	+	+	*S*. Infantis	CIP, GM, CTX, NA, TE, E, P, CTF, DO, S	*bla* CTXM-F
FCS14*	+	K/A	±	+	−	−	+	+	*S.* Infantis	CIP, GM, CTX, NA, TE, E, P, CTF, DO, S	*bla* CTXM-F
FCS17*	+	K/A	±	+	−	−	+	+	*S*. Infantis	CIP, GM, CTX, NA, TE, E, P, CTF, DO, S	*bla* CTXM-F
FCS18*	+	K/A	±	+	−	−	+	+	*S.* Infantis	CIP, GM, CTX, NA, TE, E, P, CTF, S	*bla* CTXM-F

* All isolates obtained in this study were from the same farm. FC=Farm C, S=Strain number, BR=Before receiving the birds, FI=Finisher, CIP=Ciprofloxacin, GM=Gentamicin, CTX=Cefotaxime, NA=Nalidixic acid, TE=Tetracycline, E=Erythromycin, *P=* Penicillin, CTF=Ceftiofur, S=Streptomycin, DO=Doxycycline

In XLD and SS agars, the growth of other microorganisms could be observed. These microorganisms were identified as Gram-negative bacilli through Gram staining and identified as presumptive strains of *Pseudomonas* spp., *Citrobacter* spp., *Klebsiella* spp., *Escherichia coli*, and *Proteus* spp. through biochemical tests. The latter two were the most abundant. The biosecurity characteristics, cleaning practices, disinfection, reuse of the litter, and sanitary state of the animals were recorded for each farm.

### *Salmonella* phenotypic resistance

All strains exhibited multidrug resistance (Table-1). The 16 isolates confirmed as *Salmonella* from the same farm were resistant to cefotaxime, gentamicin, penicillin, erythromycin, nalidixic acid, and ceftiofur. Of the isolates, 81.25% (13 of 16) were tetracycline resistant and the other (18.75% [3 of 16]) isolates had intermediate resistance; 93.75% (15 of 16) and 62.5% (10 of 16) had intermediate resistance to ciprofloxacin and doxycycline, respectively, and the remaining isolates were sensitive to both antibiotics. All isolates were sensitive to amoxicillin, ampicillin-sulbactam, enrofloxacin, streptomycin, colistin, and trimethoprim-sulfamethoxazole (Table-2).

**Table 2:** Results of the phenotypic resistance of the isolates of *S*. Infantis and *S*. Gallinarum obtained from the litter in poultry farms.

Antimicrobial	Conc. (μg)	Diameter of the zone of inhibition (mm)			Number and percentage of microorganisms *S.* Infantis (15) *S.* Gallinarum (1)					
	
		S	I	R	S	I	R	S	I	R
Ampicillin-sulbactam	10	≥ 15	12–14	≤ 11	15 (93.75)	-	-	1 (6.25)	-	-
Amoxicillin	10	≥ 18	14–17	≤ 13	15 (93.75)	-	-	1 (6.25)	-	-
Cefotaxime	30	≥ 8	16–32	≤ 64	-	-	15 (93.75)	-	-	1 (6.25)
Ciprofloxacin	10	≥ 31	21–30	≤ 20	-	15 (93.75)	-	1 (6.25)	-	-
Gentamicin	10	≥ 4	8	≤ 16	-	-	15 (93.75)	-	-	1 (6.25)
Erythromycin	15	≥ 23	14–22	≤ 13	-	-	15 (93.75)	-	-	1 (6.25)
Nalidyxic Acid	30	>19	14–18	< 13	-	-	15 (93.75)	-	-	1 (6.25)
Penicillin	10	≥ 22	12–21	≤ 11	-	-	15 (93.75)	-	-	1 (6.25)
Trimethoprim- sulfamethoxazole	25	>16	11–15	< 10	15 (93.75)	-	-	1 (6.25)	-	-
Tetracycline	30	>15	12–14	< 11	-	3 (18.75)	12 (75)	-	-	1 (6.25)
Ceftiofur	30	≥ 21	18–20	≤ 17	-	-	15 (93.75)	-	-	1 (6.25)
Enrofloxacin	5	≥ 21	17–20	≤ 16	15 (93.75)	-	-	1 (6.25)	-	-
Colistin	10	≥ 14	-	≤ 10	15 (93.75)	-	-	1 (6.25)	-	-
Doxycycline	30	≥ 14	11–13	≤ 10	5 (31.25)	10 (62.5)	-	1 (6.25)	-	-
Streptomycin	10	>15	12–14	≤ 11	-	-	15 (93.75)		-	1 (6.25)

R=Resistant, I=Intermediate, S=Sensitive

### Identification of ESBL resistance genes

In *Salmonella* spp. isolates resistant to cefotaxime, genes encoding ESBL were *blaCTX-M-F*, *blaCTX-M-1*, *blaCMY*, and *blaTEM*. All the isolates were positive for *blaCTX-M-F* (592 bp), but were negative for *blaCTX-M-1*, *blaCMY*, and *blaTEM* genes.

## Discussion

*S. enterica* has been determined to be responsible for salmonellosis disease in several species of production animals, including poultry. Poultry is also referred to as a vehicle for transmitting salmonellosis to humans through the consumption of broiler meat or eggs contaminated with this pathogen, thus generating economic losses and impact on public and animal health [15, 21]. *Salmonella* spp. can survive for long periods on abiotic surfaces in broiler farms [22]. One such surface is broiler litter. Litter is, therefore, considered as a source for spreading pathogens between batches of broilers [22–25]. *Salmonella* Typhimurium, *Salmonella* Enteritidis, *S*. Kentucky, and *S*. Newport are the serotypes most isolated from poultry litter [21, 22]. *S*. Infantis has been described as an emerging serotype in the poultry industry [26], as it generally does not produce symptoms in poultry. This specific serotype is, therefore, difficult to identify [27]. In Colombia, this serotype has been isolated in commercial laying hen farms in the department of Antioquia and samples of litter, feed, drinking water, cloacal swabs, cecal content, and ovaries [28]. *S*. Infantis has also been isolated in broiler carcasses and feces samples in three processing plants in the departments of Antioquia, Meta, and Cundinamarca [29]. *S*. Infantis in broiler litter has also been reported in tropical countries, such as in Ecuador and Brazil [30–32]. However, until now, the presence of *S*. Infantis has not been reported in Colombian broiler litter. Therefore, this study is the first to find *S*. Infantis in broiler litter in Colombia.

*S*. Gallinarum, the second serotype isolated in this study, generally has a low production yield and high mortality in broilers depending on the management conditions and the state of the immune system of the flock [25, 33]. In other studies, *S*. Gallinarum has been isolated from litter and cloacal swabs in Brazil and India [34, 35] and broiler organ samples in India and China [36, 37]. In Colombia, *S*. Gallinarum has been recovered mainly from carcasses [38]. Although the spread of *Salmonella* in broiler litter and farms may be due to various factors, the number of broilers on the farm is considered one of the most important determining factors [39]. Farms with small bird populations are less prone to infections than those with large populations, as higher animal density can lead to broilers experiencing more stress and having more contact with other broilers, feces, and litter, which all increase the risk of pathogen transmission, even when greater biosecurity measures are taken [4].

In addition to the threat posed by the presence of *Salmonella* in broiler litter as a source of indirect transmission to humans, a second threat is an increase in antibiotic-resistant strains used in poultry farming, as these could recirculate on farms for several production cycles and could be transferred to humans through consumption of contaminated poultry products. This could eventually lead to a failure in the treatment of salmonellosis in humans [40, 41]. In recent years, *S*. Infantis has become a public health concern in various regions worldwide due to its high resistance levels to multiple antimicrobials [42]. In fact, in this study, all *S*. Infantis and *S*. Gallinarum strains were found to be resistant to the antibiotics evaluated in the aminoglycoside group (gentamicin and streptomycin). These results were similar to those reported for *S*. Gallinarum in Nigeria and Mali in cloacal swabs and poultry organs [43, 44] and *S*. Infantis in Ecuador, England, and Japan in environmental samples from farms and poultry meat [12, 14, 45].

Likewise, in the b-lactam group, 100% resistance to cefotaxime was found, similar to the percentages reported in *S*. Infantis strains in Ecuador (87.5–99%) and Egypt, Korea, and Serbia (78.9–100%) [31, 46–48]. In *S*. Gallinarum isolates, resistance up to 85% was reported in India [49]. This resistance may be associated with cross-resistance caused by using other cephalosporins in poultry farming, such as ceftiofur [50, 51], which is an antibiotic to which all strains in this study were also resistant. Some studies have shown significant phenotypic resistance in *S*. Infantis strains to this antimicrobial in Chile (60.92–92%) [52, 53].

In addition, all *Salmonella* isolates were determined to be resistant to penicillin. These results agreed with the high resistance levels reported among other *Salmonella* serotypes, such as *S*. Enteritidis and *S*. Typhimurium, in poultry meat samples in Egypt [54]. Furthermore, all *S*. Infantis strains were sensitive to amoxicillin and ampicillin-sulbactam, in agreement with those reported in India [46] and Egypt [54]. However, results contrasted with different studies worldwide that have shown moderate resistance in *S*. Infantis and high in *S*. Gallinarum toward both antibiotics [42, 46, 55–57].

Regarding the group of quinolones, the high resistance to nalidixic acid found in this study (100%) was similar to that found in Romania and Serbia in *S*. Infantis strains from broiler meat and eggshells and that in Romania and Bangladesh in *S*. Gallinarum from healthy and sick broilers [58, 59]. Ciprofloxacin only managed to inhibit the growth of *S*. Gallinarum strain, similar to that obtained in isolates of this serotype in broiler meat in Egypt [60] and *S*. Infantis isolates from various poultry sources in Brazil [61]. The remaining 93.75% of the isolates had intermediate sensitivity to ciprofloxacin, which is similar to the sensitivity levels reported in Switzerland for *S*. Infantis from broiler meat and India for *S*. Gallinarum from internal organs of poultry [35, 62].

All isolates in this study were sensitive to enrofloxacin (100%). This sensitivity level was also described in *S*. Infantis in samples of cloacal swabs and broiler meat in Chile and Iran [52, 63]. Likewise, 100% of the strains were sensitive to colistin and trimethoprim-sulfamethoxazole. This result was in line with results obtained for *S*. Infantis in South Africa and *S*. Gallinarum in broiler embryos in China and broiler viscera in Brazil [64–66]. However, another study carried out in Argentina in broiler liver samples reported 100% sensitivity for other serovars [67].

All *Salmonella* spp. isolates were resistant to erythromycin, in line with results from South Africa in broiler samples from informal markets and results from Egypt with 83.33% of *Salmonella* strains isolated from broiler meat sourced from different supermarkets showing resistance to this antibiotic [47, 64]. Finally, the resistance levels obtained in this study for the tetracycline group differed from other studies. For example, 37.5% of the isolated *Salmonella* strains were sensitive to doxycycline. In comparison, 62.5% had intermediate sensitivity to this antibiotic, different from those reported for *S*. Infantis in South Africa for samples of broiler meat (50% resistance) and those reported in Indian farms for environmental samples (100% resistance). In contrast, resistance levels to tetracycline were similar to those found in Brazil and Japan in meat samples, which were as high as 84.8% and 96.5%, respectively [67, 68].

Antibiotic resistance in *Salmonella* spp. generally depends on the acquisition of resistance genes from their environment [69]. For b-lactams, *blaCTX-M* genes are commonly related to resistance to cephalosporins [70]. This gene has numerous variants, such as *blaCTX-M-F*, detected in all isolates with phenotypes resistant to cefotaxime. These results were in line with the data reported in Brazil and Ecuador in *S*. Infantis isolates from broiler ceca [8, 14]. Other genes associated with the resistance of *Salmonella* spp. b-lactams are *blaTEM* and *blaCMY* [71], which are genes also analyzed in this study but were not detected in any isolated *Salmonella* samples. However, *blaTEM* has been reported in various serotypes from poultry environments in India (*S*. Typhimurium), Colombia (*S*. Newport, *S*. Paratyphi B, and *S*. Manhattan), and China (*S*. Gallinarum) [9, 46, 72, 73]. The *blaCMY* gene was present with the *blaCMY-2* variation in *S*. Infantis in Japan and Egypt in broiler meat samples [11, 69]. These resistance genes in *Salmonella* spp. from poultry samples represent a significant risk to public health, as they can be transferred horizontally through mobile elements, such as plasmids [12]. Through plasmids, genes could not only reach hosts through poultry products contaminated with resistant bacteria but also through mechanical and biological vectors, such as flies and *Alphitobius diaperinus*, found in high densities in the litter analyzed in this study [70, 74].

## Conclusion

Serotypes *S*. Infantis and *S*. Gallinarum were detected in the litter of broilers in Tolima. These serotypes increase the risk of the spread of *Salmonella* on farms and are a possible source of transfer of the pathogen and its genes to humans. All strains resistant to antibiotics are ranked by the World Health Organization as drugs of highest priority for treating diseases in humans and identified by the World Organization for Animal Health as critically important veterinary antimicrobial agents. Furthermore, all isolates carried the *blaCTXM-F* gene associated with resistance to cefotaxime. These results showed that *Salmonella* serotypes are changing in poultry litter in Colombia.

Future studies are expected to intensify the collaboration with and participation of the farms in the Tolima region to increase the number of samples. Furthermore, this study was expected to serve as a base to characterize the state of resistance of pathogenic microorganisms and identify the *Salmonella* serotypes present in litter in different communities dedicated to raising broilers in surrounding areas. This would allow for developing prevention and control plans for these microorganisms. The first avenue of future research would be to study if results were similar in other regions in Colombia among small producers. The second avenue would be to distinguish between different production systems (small scale and industrial) in the study on *Salmonella* in broiler litter.

## Data availability

Supplementary data can be available from the corresponding author on a reasonable request.

## Authors’ Contributions

MV and MAO: Designed the study and revised the manuscript. MAB and MPL: Collected data, performed the experimental work, and drafted the manuscript. All authors have read and approved the final manuscript.
